# Integrating optical coherence tomography with gravimetric and video analysis (OCT-Gravimetry-Video method) for studying the drying process of polystyrene latex system

**DOI:** 10.1038/s41598-018-30914-8

**Published:** 2018-08-28

**Authors:** Hao Huang, Yongyang Huang, Willie Lau, H. Daniel Ou-Yang, Chao Zhou, Mohamed S. El-Aasser

**Affiliations:** 1 Department of Chemical and Biomolecular Engineering, Lehigh University, Bethlehem, Pennsylvania 18015 USA; 2 Emulsion Polymers Institute, Lehigh University, Bethlehem, Pennsylvania 18015 USA; 3 Department of Electrical and Computer Engineering, Lehigh University, Bethlehem, Pennsylvania 18015 USA; 4 Beijing Oriental Yuhong Waterproof Technology Co., Ltd, Beijing, 100123 China; 5 State Key Laboratory of Special Functional Waterproof Materials, Beijing, 101309 China; 6 Department of Physics, Lehigh University, Bethlehem, Pennsylvania 18015 USA; 7 Department of Bioengineering, Lehigh University, Bethlehem, Pennsylvania 18015 USA

## Abstract

Latex, an aqueous dispersion of sub-micron polymer particles, is widely used as polymer binder in waterborne coatings and adhesives. Drying of a latex is inhomogeneous, during which the spatial distribution of particles is non-uniform and changes with time, usually resulting in a compromise of the integrity of a dried film. To study drying inhomogeneity of latex, we developed a system integrating optical coherence tomography (OCT) with gravimetric and video analysis (OCT-Gravimetry-Video method) to non-destructively monitor the drying process of non-film-forming latexes consisting of hard polystyrene spheres over time. OCT structural and speckle images of the latex’s internal structure show the packing process of particles, the detachment of latex and the formation of apparent shear bands in cross-sectional views. Video recordings show the formation of cracks and the propagation of the drying boundary in the horizontal direction. The drying curve, measured by gravimetry, shows the drying rate and the water content of the latex at each drying stage. Furthermore, we find that the particle size affects packing and cracking phenomena remarkably. The OCT-Gravimetry-Video method serves as a general and robust approach to investigate the drying process of waterborne latex system. This method can be employed for fundamental studies of colloids and for evaluations of industrial latex products.

## Introduction

Latex, or polymer colloids, is a colloidal system of polymer particles suspended in aqueous medium. Particle sizes in latexes range from tens to hundreds of nanometers. Latex is normally produced by emulsion polymerization^1^, with a global demand of more than 10 million metric tons (around 30 billion US dollars) per year^2^. By drying a pool of latex with a specific thickness (typically from tens to hundreds of microns), a polymer film can be formed to cover a material surface for protective, aesthetic and adhesive purposes. In some applications, such as architectural coatings and roofing, the thicknesses of the dried films range from tens of microns to as much as several millimeters. Waterborne latex systems are widely used in coating and adhesive products, as well as inks, cosmetics, pharmaceuticals, vehicle coatings, paper coatings, carpet backing, etc. During its drying process, the latex can suffer from “drying inhomogeneity”, in which the spatial distribution of latex particles is non-uniform and changes with drying time, usually resulting in the formation of defects^3^. In the horizontal direction, the “coffee ring” effect^4^ packs particles and macromolecules near the circumferential edge, causing short open time for film leveling^5–7^; in the vertical direction, the “snow plow effect”^8,9^ packs particles and macromolecules near the top surface, causing skin layer formation^10,11^; with the loss of water, interfacial tensions (or capillary forces) between particles build up the internal compressive stresses that tend to shrink the volume of the latex film, and the restriction by the substrate which counteracts the shrinkage leads to the formation of cracks when the polymer’s glass-transition temperature (*T*_*g*_) is higher than the room temperature (for which particles lack deformation)^12–14^.

Theoretical models on the drying inhomogeneity were established to simulate the time-evolution of non-uniform distribution of simple hard spheres^5,8,10,13^. More recent studies show that interactions between particles, water-soluble molecules and interfacial tensions all influence the spatial distribution of latex particles^9,15,16^. Also, experimental approaches were exploited to analyze the drying inhomogeneity. Gravimetric analysis and videography, which record global water loss and film appearance respectively, are most common methods^7,17^. Nuclear magnetic resonance profiling (NMR)^11^, Raman microspectroscopy^18^ and infrared microscopy^19^ can non-destructively measure the distribution of water concentration inside the latex, but lack the resolution to see particles. With microns or sub-micron resolutions, environmental scanning electron microscopy (ESEM), ellipsometry, atomic force microscopy (AFM) were used to detect particles’ packing process, during which particles gathered with their Brownian motions restricted^9,20–22^. However, ESEM, ellipsometry and AFM can only observe the top surface of the latex with a low penetration of <50 *μ*m. Cryogenic scanning electron microscopy (Cryo-SEM) was used to view the cross-section of latex and observed particles’ packing, but with a concern of damaging the sample^23^. Diffusing-wave spectroscopy (DWS) and laser speckle imaging (LSI) characterize the mobility of latex particles near the top surface at different drying stages^9,24–26^. LSI can be used to map the drying inhomogeneity in the horizontal imaging plane; and it is also sensitive to structure and dynamics at different depths within the sample and obtains valuable information, such as the delamination of coating from the substrate^26^. However, LSI cannot provide depth-resolved imaging of the drying dynamics within the latex (e.g. film formation, snow plow effect, etc.) with high spatial resolution.

Optical coherence tomography (OCT), a non-destructive and non-invasive optical imaging technique, is capable of obtaining cross-sectional images inside a sample with a penetration depth up to 1–2 mm using near-infrared light illumination^27–30^. OCT relies on low-coherence interferometry to detect back-scattered light signals within the sample and provides a reconstructed image of scattered light intensity with micron-scale resolutions. In the biomedical field, OCT has been widely adopted for opthalmic^31^ and cardiac imaging^32,33^. Although OCT was used to track motions of micron-size particles in drying colloid^34,35^, OCT has not yet been used for investigating the mobility of latex’s sub-micron particles or the internal microstructures of latex at different drying stages.

In this article, we present our studies on the drying process of polystyrene latexes at room temperature and humidity. The model latexes contain polystyrene (PS) particles with diameters of 125 nm and 53 nm (designated as “L latex” and “S latex”, respectively). These particles do not deform during drying because of high *T*_*g*_ of PS (>100 °C). To non-destructively monitor the drying process of the model latex over time, here we develop an integrated system, which includes simultaneous OCT imaging, gravimetric measurement, and video recording. This system, which we call the “OCT-Gravimetry-Video” method, is able to characterize both macroscopic and microscopic features of the polystyrene latex. Specifically, time-lapse OCT imaging shows not only the thickness change and the latex’s detachment off the substrate, but also the formation of apparent shear bands in cross-sectional views. In addition, a speckle contrast analysis^25^ is implemented to differentiate mobilities of particles in the latex in order to visualize the dynamic packing process of particles in the vertical direction (“snow plow effect”). The video camera, which records latex’s macroscopic appearance from the top view, shows the drying boundary and cracks that propagate along the horizontal surface. Gravimetry, using a digital balance, measures the global drying curve, from which the drying rates and the water contents in latex at different times are derived. The drying curves are correlated with the different drying stages observed in OCT images and video photos. Comparing characterization results of L and S latex systems, we observe that particle size affects the packing and cracking phenomena remarkably during the drying process of latex.

## Results and Discussions

### Global drying and local drying characterized by OCT-Gravimetry-Video method

**Figure 1:**
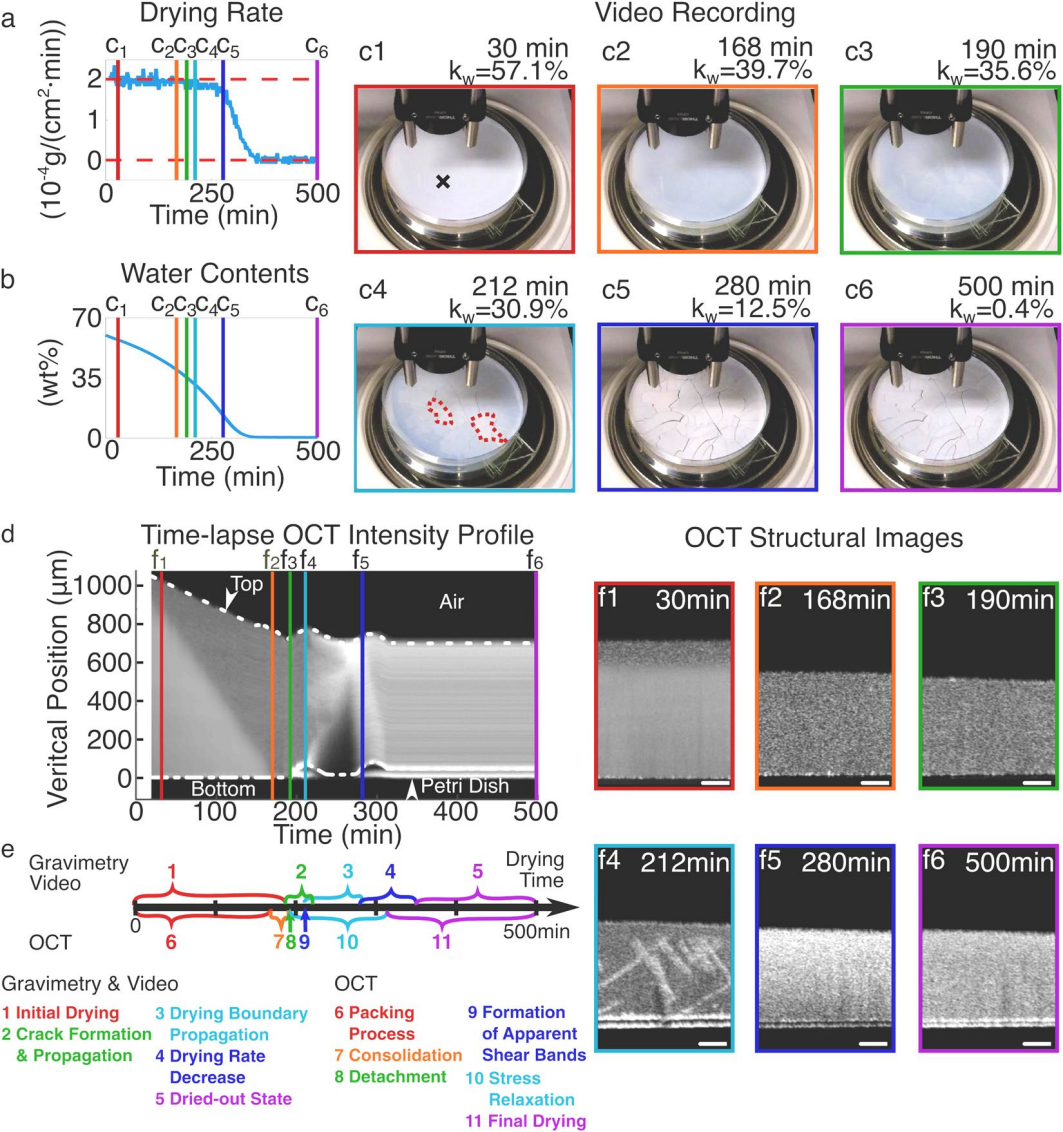
OCT-Gravimetry-Video characterization of the drying process of L latex particles (diameter ∼125 nm). (**a**,**b**) Curves of the global drying rate (*dw*(*t*)/*dt*) and the water content in latex (*k*_*w*_(*t*)) derived from gravimetric measurement. (**c1–c6**) video photos at 30, 168, 190, 212, 280 and 500 min. (**d**) Time-lapse OCT intensity profile, derived from 2D OCT structural images at different times. (**e**) Drying states from the start of drying to the end of being fully dried, defined by the Gravimetry-Video characterization (above the timeline) and by the OCT imaging (below the timeline). (**f1–f6**) 2D OCT structural images at 30, 168, 190, 212, 280 and 500 min. Scale bars in (**f1–f6**): 200 *μ*m. Black cross in (**c1**): OCT scanning location. Red dotted circles in (**c4**): drying boundary.

**Figure 2:**
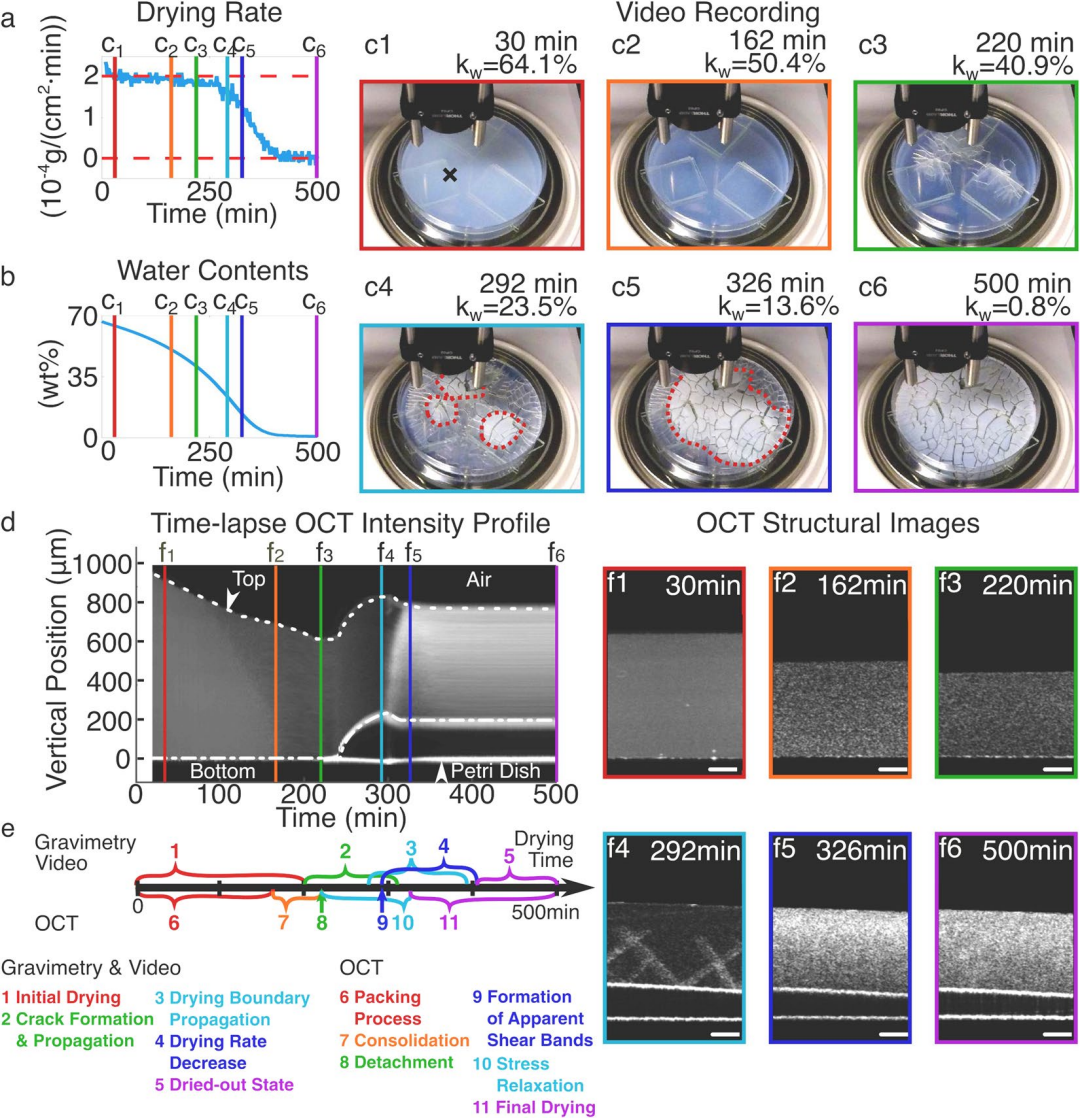
OCT-Gravimetry-Video characterization of the drying process of S latex particles (diameter ∼53 nm). (**a**,**b**) Curves of the global drying rate (*dw*(*t*)/*dt*) and the water content in latex (*k*_*w*_(*t*)) derived from gravimetric measurement. (**c1–c6**) video photos at 30, 162, 220, 292, 326 and 500 min. (**d**) Time-lapse OCT intensity profile, derived from 2D OCT structural images at different times. (**e**) Drying states from the start of drying to the end of being fully dried, defined by the Gravimetry-Video characterization (above the timeline) and by the OCT imaging (below the timeline). (**f1–f6**) 2D OCT structural images at 30, 162, 220, 292, 326 and 500 min. Scale bars in (**f1–f6**): 200 *μ*m. Black cross in (**c1**): OCT scanning location. Red dotted circles in (**c4)** and (**c5**): drying boundary.

Figs 1 and 2 (as well as Supplementary Videos 1 and 2) present the time-lapse OCT-Gravimetry-Video measurements of the drying processes of polystyrene latexes (with the initial wet latex thickness of ∼1 mm). The OCT imaging system is configured to have a vertical resolution of ∼4.3 *μ*m and a horizontal resolution of ∼14 *μ*m (see Materials and Methods section). Two polystyrene latexes, consisting of larger particles (L latex) and smaller particles (S latex) respectively, were used. The local internal microstructure at the OCT scanning spot, the global drying curve and the top-view visual appearance of these two latexes were characterized simultaneously. Characterization results from different measurements were correlated to provide insights of drying inhomogeneities in each state of the drying process. During drying of those two latexes, the room temperature and humidity were measured as 26 ± 1 °C and 45 ± 1% RH, and the corresponding drying rate was (2.0 ± 0.1) × 10^−4^ *g*/(*cm*^2^⋅*min*).

#### Polystyrene latex with larger particles (L latex, particle diameter: ∼125 nm)

For L latex, from 0 min to ∼186 min, the video recording shows that the top surface of the latex was smooth, and its color changed from opaque white to transparent blue (Figs 1c1–c2). The color change was because the interstices between particles decreased as the water evaporated so that the short-wavelength light had stronger scattering compared to long-wavelength light due to Rayleigh scattering^7,36^. This drying state is referred to as the “initial drying” state (#1 in Fig. 1e). In this state, the measured global drying rate was constant (Fig. 1a). The drying rate was the same as that of de-ionized (DI) water ((2.0 ± 0.1) × 10^−4^*g*/(*cm*^2^⋅*min*)) measured under the same room condition. The thickness of the latex (i.e. vertical distance between the top and the bottom of latex) decreased linearly with time from ∼1100 *μ*m to ∼720 *μ*m, shown in the time-lapse OCT intensity profile (from 0 min to near the line of *f*_3_ in Fig. 1d). This agrees with the measured constant drying rate.

After ∼186 min, the video shows that cracks appeared in the middle of the Petri dish and propagated to the dish wall until ∼220 min (referred to as the “crack formation and propagation” state, #2 in Fig. 1e). During this time, the L latex was broken up into pieces (Figs 1c3-c4 and Supplementary Video 1). At ∼190 min, when cracks appeared at the OCT scanning location (Fig. 1c3), the time-lapse OCT intensity profile shows the latex detached and moved up off the dish substrate (Fig. 1d, the “detachment” state in Fig. 1e). From ∼186 min to ∼220 min, the water content of the whole latex (*k*_*w*_) decreased from 36.4 wt% to 29.1 wt% (Fig. 1b). Since the density of polystyrene (1.04 *g*/*cm*^3^) is quite close to that of water (1 *g*/*cm*^3^), *k*_*w*_ can represent the void fraction of the interstices between the latex particles that are occupied by water. Before the detachment of the latex, the substrate exerted tensile stress to the latex to counteract its volume shrinkage caused by the internal compressive stress (due to the interfacial tensions or capillary forces between particles). Such tensile stress induced the formation of cracks later on, even though there were more than 30% of water inside the latex^13,37^.

From ∼210 min to ∼278 min, the video shows that a clear drying boundary appeared in the middle of the Petri dish and propagated to the dish wall (Fig. 1c4 and Supplementary Video 1). The drying boundary separated the enclosed white drier region from the outside blue wetter area, with the enclosed region expanding over time. The whitening of the enclosed region (Fig. 1c4) can be attributed to air infiltration into the latex, causing a change of its scattering property. This drying state was named the “drying boundary propagation” state (#3 in Fig. 1e). Although 13.1 wt% water was in the latex at ∼278 min, the latex remained white and barely changed afterwards in the video (Fig. 1c6). Since the structure of latex was porous, packed particles did not inhibit drying. Ideally, if drying was uniform everywhere on the horizontal surface, the drying rate should drop abruptly from free water evaporation to zero at the point in time when all water evaporated. However, it took ∼72 min for the drying rate to decrease from $\sim 1.7\times {10}^{-4}g/(c{m}^{2}\cdot min)$ at ∼276 min to nearly 0 at ∼348 min (Fig. 1a, corresponding to the “drying rate decrease” state, #4 in Fig. 1e). The reason is that the horizontal drying inhomogeneity causes the dryings at different positions to stop at different times. The period of “drying rate decrease” state (∼72 min) is close to that of “drying boundary propagation” state (∼68 min). After ∼348 min, the latex was completely dried out due to nearly zero drying rate and zero water content.

Interestingly, as the drying boundary reached the OCT scanning location (i.e. the black cross in Fig. 1c1) at ∼212 min, the 2D cross-sectional OCT image of Fig. 1f4 shows the cross-shaped lines with high scattered light intensity. Those lines developed at ∼30–40° to the direction of drying and happened for both L latex (Fig. 1f4) and S latex (Fig. 2f4), resembling the structure of shear bands that were observed and investigated by Yang *et al*.^38^ and Kiatkirakajorn *et al*.^39^ with the drying polystyrene and silica colloids. Thus, in this article, the cross-shaped lines in Figs 1f4 and 2f4 are called “apparent shear bands”, since their real structures haven’t been confirmed yet in our experiments. And the “formation of apparent shear bands” state (#9 in Fig. 1e) occurred at ∼212 min. We propose that the apparent shear bands are the dislocations of the packed latex particles where air infiltrates, which are induced by the internal compressive stresses (induced by the interfacial tensions or capillary forces between particles) along the vertical direction, although the water content of the latex remains high (*k*_*w*_ = 30.9 wt% at ∼212 min). The apparent shear bands lasted for ∼68 minutes and disappeared at ∼280 min (Fig. 1f5). Between ∼190 min and ∼314 min, the time-lapse OCT intensity profile shows the latex moved up and down with the formation of cracks and apparent shear bands. During that period, the latex film kept relaxing its internal stresses. Thus, this drying state was called the “stress relaxation” (#10 in Fig. 1e). After ∼314 min, the latex at the OCT scanning location barely changed and remained detached (Fig. 1f6 and Supplementary Video 1), corresponding to the “final drying” state (#11 in Fig. 1e).

#### Polystyrene latex with smaller particles (S latex, particle diameter: ∼53 nm)

For S latex, from 0 min to ∼200 min, the video shows that the top surface of latex was smooth (Figs 2c1-c2, and Supplementary Video 2), corresponding to the “initial drying” state (#1 in Fig. 2e). The color was bluer and more transparent as compared to the L latex because smaller interstices between smaller particles lead to stronger scattering of short-wavelength light than scattering of long-wavelength light^7,36^. The drying rate was the same as that of DI water (Fig. 2a). Starting from ∼200 min, cracks appeared and propagated to the Petri dish wall until ∼310 min (Figs 2c3-c4 and Supplementary Video 2), corresponding to the “crack formation and propagation” state (#2 in Fig. 2e). When the cracks reached the OCT scanning location at ∼220 min, the time-lapse OCT intensity profile shows the start of latex’s detachment away from the substrate (Fig. 2d, the “detachment” state, #8 in Fig. 2e). The water content of S latex changed from 44.5% at 200 min to 18.3% at 310 min.

From ∼276 min to ∼392 min, the video shows a clear drying boundary emerged and propagated to the Petri dish wall (Figs 2c4-c5 and Supplementary Video 2), corresponding to the “drying boundary propagation” (#3 in Fig. 2e). After ∼392 min, the latex remained white and barely changed (Fig. 2c6 and Supplementary Video 2). From ∼292 min to ∼406 min, which took ∼114 min, the drying rate decreased from $\sim 1.7\times {10}^{-4}g/(c{m}^{2}\cdot min)$ to nearly 0 (Fig. 2a), corresponding to the “drying rate decrease” state (#4 in Fig. 2e). The period of “drying rate decrease” state (∼114 min) is close to that of “drying boundary propagation” state (∼116 min). When the drying boundary reached the OCT scanning location at ∼292 min, the 2D OCT structural image shows the formation of apparent shear bands along the vertical direction (Fig. 2f4, the “formation of apparent shear bands” state, #9 in Fig. 2e). The apparent shear bands lasted for ∼34 minutes and disappeared at ∼326 min. Between ∼220 min and ∼326 min, the time-lapse OCT intensity profile shows the latex moved up and down (the “stress relaxation” state, #10 in Fig. 2e). Afterwards, the latex at the OCT scanning location remained unchanged and detached (Fig. 2f6, the “final drying” state, #11 in Fig. 2e).

By comparing the crack patterns of L latex (Fig. 1c6) and S latex (Fig. 2c6), the broken-up pieces’ areas of S latex (27.4 ± 24.1 mm^2^) are generally smaller than those of L latex (66.8 ± 60.3 mm^2^). Such difference can be attributed to the different compressive stresses, which are inversely proportional to the particle size^3,13^. The smaller the particles are, the higher the internal stresses are, therefore more cracks are needed to release the accumulated stress energy, and the smaller the areas of the broken-up pieces are.

### Visualization of particles’ packing process using OCT

**Figure 3:**
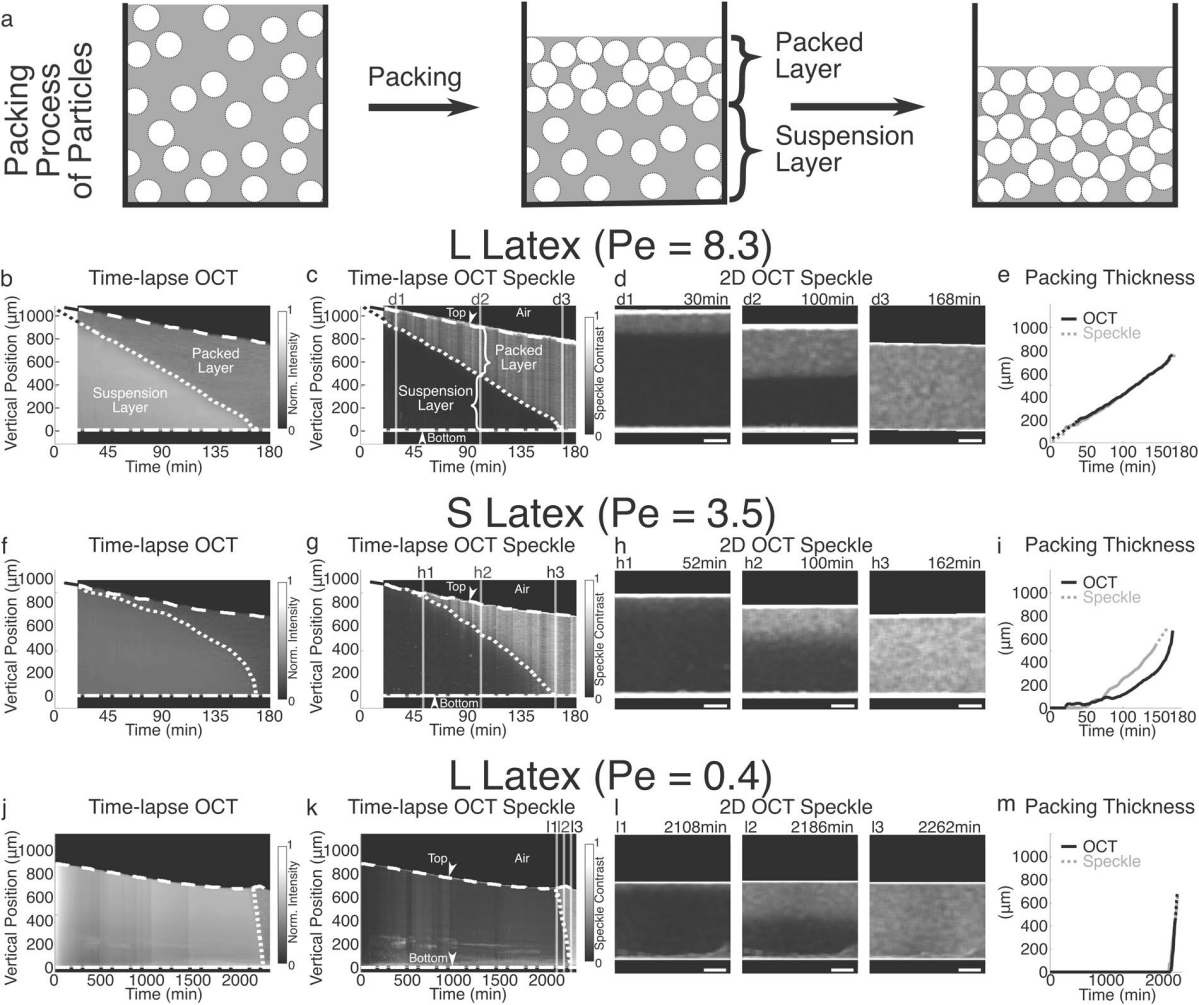
Visualization of particles’ packing process using OCT. (**a**) Schematic illustration of the packing process of particles. (**b**,**f**) Time-lapse OCT intensity profiles of L and S latexes, respectively. (**c**,**g**) Time-lapse OCT speckle profiles of L and S latexes, respectively. The domain boundary curves separating the upper packed layer and the lower suspension layer, shown as the dotted curves inside the latexes in (**b**,**f**,**c**,**g**), were determined according to the scattered light intensity in (**b**,**f**) or speckle contrast values in (**c**,**g**) (see Materials and Methods section for details). (**d1–d3**) 2D OCT speckle images at ∼30, ∼100 and ∼168 min of L latex. (**h1–h3**) 2D OCT speckle images at ∼52, ∼100 and ∼162 min of S latex. (**e**) L latex: packed layer thickness changes as a function of time derived based on light intensity or speckle analysis, respectively. (**i**) S latex: packed layer thickness changes as a function of time derived based on light intensity or speckle analysis, respectively. The drying rates of both L and S latexes were the same ((2.0 ± 0.1) × 10^−4^ *g/(cm*^*2*^ *min))*, with the calculated *Pe’s as* 8.3 and 3.5, respectively. (**j**–**m**) Time-lapse OCT intensity profile, time-lapse OCT speckle profile, 2D OCT speckle images at different times, and packed layer thickness curves of L latex at a slow drying rate of $0.097\times {10}^{-4}g/(c{m}^{2}\cdot min)$ with *Pe* = 0.4. The drying rate was reduced by covering the sample with the Petri dish lid. Scale bars in (**d**), (**h**) and (**l**) are 200 *μ*m.

The dynamic process of particles’ packing can be visualized in the time-lapse OCT images of both L and S latexes. Fig. 3a illustrates the packing process of particles in the vertical direction, known as the snow plow effect. At the beginning, particles in latex are separated by large distances and move freely in space. As water continues to evaporate, the latex becomes divided into two domains in the vertical direction: in the upper domain (i.e. “packed layer”), particles are in contact with each other with smaller interstices and low mobility; in the lower domain (i.e. “suspension layer”), particles remain separately suspended and freely move in water with larger distance between their surfaces. At the end of the packing process, all particles are packed and their motions are restricted.

Figs 3b and f show the time-lapse OCT intensity profiles of L and S latexes, respectively. Within the first 180 min, a domain boundary curve was observed in the latex’s cross-section, which is shown as dotted curves in Figs 3b and f, respectively. The curve separated the upper domain with lower scattered light intensity and the lower domain with higher scattered light intensity, which suggested that these two domains had different internal structures. To verify it, we performed speckle contrast analysis on 2D OCT structural images and time-lapse OCT intensity profiles (see Materials and Methods section for details). The mobility of particles in these two domains can be clearly differentiated from the OCT speckle contrast images. The higher the speckle contrast (*K*_*s*_, calculated by Equation (6)) is, the lower the mobility of particles is. Figs 3c and g show the time-lapse OCT speckle profiles of L and S latexes, respectively. The domain boundaries can be easily identified based on speckle contrast (shown as dotted curves in Figs 3c and g). Above the boundary, *K*_*s*_ is high indicating low particle mobility; below the boundary, *K*_*s*_ is low indicating high particle mobility. Such domain boundaries can be also observed in 2D OCT speckle images (Figs 3d1–d3,h1–h3). These results of the speckle contrast analysis indicated that the upper and lower domains of the latex in both time-lapse OCT intensity and speckle profiles are the packed layer and the suspension layer, respectively. Supplementary Video 3 and 4 further exhibit the evolution of the packed and suspension layers in both L and S latexes, respectively. It should be noted that the data during the initial 20 min in the OCT profiles (i.e. Figs 3b,c,f,g) were missing, because the time was spent on weighing and spreading the latex in Petri dish, adjusting the OCT system, and synchronizing the OCT-Gravimetry-Video setup. An additional OCT experiment of the packing process of L latex during the initial 30 min was performed (by quickly pouring the latex in Petri dish and put under the OCT objective) and shown as Supplementary Video 5.

We further quantified the thickness change of the packed layer to show the difference of packing processes between L and S latexes. The packed layer thickness of the latex was determined by the distance between its top surface and its domain boundary curve, derived based on either light intensity (Fig. 3b and f) or speckle contrast (Figs 3c and g) images. Results are shown in Fig. 3e for L latex and Fig. 3i for S latex, respectively. For L latex, the packed layer thickness was observed to increase linearly over time, and the thickness curve extrapolates to ∼0 *μ*m at 0 min (when the latex was cast in a Petri dish). Supplementary Video 5 also shows that the packing process started from the beginning (∼0 min). The packing process ended at ∼168 min, according to the speckle analysis. Thus, we referred to the period between 0 min and ∼168 min as the “packing process” state (#6 in Fig. 1e). Packed layer thicknesses quantified by the intensity and the speckle contrast analysis overlapped very well for L latex (Fig. 3e). For S latex, the packed layer was not evident during the initial ∼48 min, where the packed layer might be too thin to be resolved by OCT (with a resolution of ∼4.3 *μ*m). After that, the packed layer thickness began to increase quickly over time. The packing process ended at ∼162 min. However, we observed that, although the two curves determined by the intensity and the speckle contrast methods had the same growing trend, they did not completely overlap with each other for S latex (Fig. 3i). This is because the intensity contrast between packed layer and suspension layer in Fig. 3f is so small that the domain boundary cannot be clearly defined. On the other hand, the speckle analysis is more sensitive to particle’s mobility, so that it can provide higher contrast to define the domain boundary more accurately in Fig. 3g.

Besides the difference of the packed layer thickness curves between L and S latexes, we also observed other packing process differences between these two samples. One difference is the sharpness of the packing/suspension domain boundary (or the width of domain boundary). In OCT speckle images, L latex has a sharper domain boundary (Fig. 3c) than S latex (Fig. 3g); in another word, the transition of speckle contrast (*K*_*s*_) from high value of packed layer to low value of suspension layer is more instant for L latex than that for S latex. Routh and Zimmerman^8^ predicted a scaling relation for simulating the particles’ packing process based on the Peclet number (*Pe*), which is the rate of water evaporation divided by the rate of particles’ diffusion. In the dilute limit where Stokes-Einstein equation determines the diffusion coefficient of particles, *Pe* can be written as^11^:

1 $$Pe=\frac{6\pi \mu {R}_{0}{H}_{0}\dot{E}}{{k}_{B}T}$$

where *μ* is the water viscosity, *R*_0_ is the particle radius, *H*_0_ is the initial thickness of latex, $\dot{E}$ is the water level receding velocity (the ratio of drying rate over water density), *k*_*B*_ is the Boltzmann constant and *T* is the temperature. According to their model, the higher the ratio of water evaporation rate over particles’ diffusion rate (larger *Pe*) is, the sharper the packing/suspension domain boundary is (or the narrower the domain boundary width is), which was also shown by the NMR profiles of water distribution within latex films^40^. In our experiments, *Pe* for L and S latexes were calculated as 8.3 and 3.5, respectively. This explains the difference of the boundary sharpness. Note that, the simulation results by Routh and Zimmerman did not consider interactions between particles. In reality, electrostatic or steric repulsive interactions, which are related to chemical groups on particles’ surface, surfactants and salts, can dramatically change the diffusion coefficient of particles and the Peclet number^1,9^.

Another difference of packing process is the volume fraction of particles (*ϕ*_*p*_) when they get packed. As shown in Figs 1d and 2d at the OCT scanning location, due to the constant drying rate, the thickness of latex (*H*, vertical distance between the top and the bottom of latex) decreased linearly from the start (0 min), to the end of packing process (f2 in Figs 1d and 2d), and even until the start of detachment or stress relaxation (f3 in Figs 1d and 2d). The period between f2 and f3 is called the “consolidation” state (#7 in Figs 1e and 2e), during which the particles become more densely packed. By equating the volume of particles in latex at 0 min and that at a point of time, *ϕ*_*p*_ can be calculated as:

2 $$H{\varphi }_{p}={H}_{0}{\varphi }_{p0}$$

where *ϕ*_*p*0_ is the initial volume fraction of particles, which is approximately equal to the initial solid weight content in latex (*k*_*s*0_) which is 40.11% for L latex and 33.43% for S latex (see Materials and Methods section) due to the similar density of polystyrene versus water. Based on the measurements of thicknesses in Figs 1d and 2d, *ϕ*_*p*_ at the end of packing (*ϕ*_*p*,*f*2_) is 56.3% for L latex and 48.6% for S latex. Both *ϕ*_*p*,*f*2_ values are lower than 74 vol% (*ϕ*_*p*_ of the densest regular packing which is either hexagonal close-packing or face-centered cubic packing) or 64 vol% (*ϕ*_*p*_ of random close packing)^41^, indicating that particles at the end of packing are either randomly loose packed or spaced apart. *ϕ*_*p*_ at the end of consolidation (*ϕ*_*p*,*f*3_) increases to 61.4% for L latex and 54.7% for S latex, which is still below 64 vol%. Interestingly, for both *ϕ*_*p*,*f*2_ and *ϕ*_*p*,*f*3_, the particles’ volume fraction of L latex is about 7% higher than that of S latex, which may be related to the differences in the packing geometry and in the distance between particles that need further studies.

Another experiment with *Pe* = 0.4 was performed by slowing down the drying rate of L latex by about 20 times (see the OCT profiles in Figs 3j–m and Supplementary Fig. 1). As expected by Routh and Zimmerman’s model, with a low $Pe < 1$, the packed layer was not visible initially. The packing process started at ∼2108 min and finished within ∼154 min, during which only a little amount of water evaporated with *k*_*w*_ changing from 48.4% (at ∼2108 min) to 47.0% (at ∼2262 min). Based on the measurements of thicknesses in Supplementary Fig. 1, *ϕ*_*p*_ at the end of packing (*ϕ*_*p*,*f*2_) at 2262 min is 53.0%, and *ϕ*_*p*_ at the end of consolidation or the start of detachment (*ϕ*_*p*,*f*3_) at ∼2980 min is 56.4%. Interestingly, both values are lower than *ϕ*_*p*,*f*2_ = 56.3% and *ϕ*_*p*,*f*3_ = 61.4% of L latex that was dried normally in Fig. 1d. This is counterintuitive to the sense that slower packing process makes the packing more dense with larger *ϕ*_*p*_, and thus needs to be investigated in future. The packing process of latex particles should be far more complicated than simply stacking solid spheres with Brownian motions. In order to precisely predict the profile of particles’ distribution during drying, the particles’ size and interaction, the void fraction of packing state and even the drying rate need to be taken into account in future studies.

## Conclusions

Drying inhomogeneity is a universal phenomenon in the latex that the spatial distribution of latex particles is non-uniform and changes with time, usually resulting in the formation of drying defects in the latex film. In this article, a new method by combining OCT with gravimetric measurement and video recording (“OCT-Gravimetry-Video” method) is developed to characterize the drying process of polystyrene latex systems. OCT can image the drying structure inside the latex with microns’ resolution, including the local drying inhomogeneities in the vertical direction. Packing of particles, latex detachment, and apparent shear bands are observed. The gravimetric measurement shows the drying rate and water content as a function of drying time according to the measured drying curve. The video takes time-lapse photos of the latex from the top view. Both the gravimetry and the video can show drying inhomogeneities in the horizontal direction, including the propagation of the drying boundary and cracks. Characterization results from OCT, gravimetry and video were correlated in each state of the drying process. Experiments on two polystyrene latexes with ∼125 nm and ∼53 nm particle diameters show different behaviors of the packing process and the formation of cracks. OCT-Gravimetry-Video method is of great value since it offers comprehensive information about the dynamic drying process to study drying inhomogeneities of waterborne latex systems. This method can be used for fundamental studies of colloidal system and for evaluating industrial latex products.

## Materials and Methods

### Latex preparation and characterizations

Polystyrene (PS) latexes were synthesized by semi-batch emulsion polymerization^1^. The polymer composition of PS particles is 99 wt% PS with 1 wt% polymethacrylic acid. The latexes were neutralized with sodium hydroxide until the pH reached 8, so that the acid groups on particles’ surface are ionized to help stabilize the particles. Styrene monomer and methacrylic acid monomer for synthesis are purchased from ACROS Organics (USA) and Sigma-Aldrich (USA), respectively. The surfactant used for stabilizing particles is sodium lauryl sulfate (SLS), purchased from Alfa Aesar (USA). By adjusting the amount of SLS added during synthesis, the size of the latex particles can be controlled. Characterizations of the PS latex include glass-transition temperature (*T*_*g*_) of the polymer by Q2000 differential scanning calorimetry (TA Instruments, USA), hydrodynamic particle diameter by dynamic light scattering (DLS) – ALV/CGS-3 Goniometer System (ALV GmbH, Germany), Zeta potential by Zetasizer Nano ZS (Malvern Panalytical, UK), and solid content (*k*_*s*0_) by taking the weight ratio of the dried latex (after being dried at 95 °C for 24 h) over the original latex. Two PS latexes are used in our experiments – “L latex” with larger particle size and “S latex” with smaller particle size, with particles’ diameters of ∼125 nm (standard deviation = 23 nm) and ∼53 nm (standard deviation = 7 nm), respectively. The Zeta potentials of the particles were measured as −84 mV for L latex and −72 mV for S latex, respectively, in a dilute sodium bicarbonate solution with pH = 8. The *T*_*g*_’s of L and S latex polymers are 107 °C and 106 °C, respectively. *k*_*s*0_ of L latex and S latex are 40.11 wt% and 33.43 wt%, respectively. Since the density of polystyrene (1.04 *g*/*cm*^3^) is quite close to that of water (1 *g*/*cm*^3^), *k*_*s*0_ can represent the volume fraction of particles in latex (*ϕ*_*p*0_).

### OCT-Gravimetry-Video method

**Figure 4:**
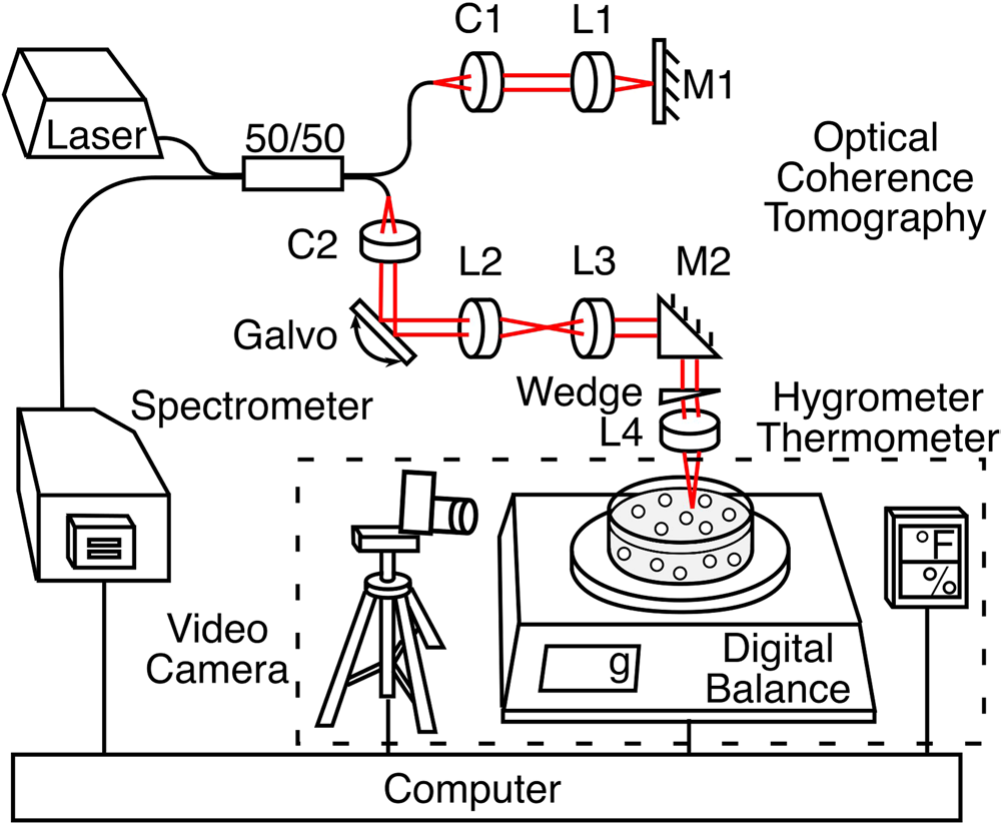
Schematic diagram of OCT-Gravimetry-Video setup.

Fig. 4 shows the schematic diagram of OCT-Gravimetry-Video setup, integrating an OCT imaging system, a digital balance, and a video camera. OCT acquires cross-sectional images of the latex with micron resolutions by detecting back-scattered light intensity via low coherence interferometry. The digital balance measures the water mass loss of the latex over time. The video camera records changes of the overall appearance of the latex under white light. Drying of a latex was conducted under room conditions.

For OCT imaging, a superluminescent diode (SLD, SLD1325, Thorlabs, USA) with a central wavelength of 1320 nm and a spectral range of 110 nm was used. The light was transmitted through a 50/50 optical coupler, with 50% power to the sample arm and the other half of power to the reference arm. A 2D galvanometer (Galvo, GVS 002, Thorlabs) was utilized on the sample arm to scan the light beam on the latex along the horizontal direction. A lens with a focal length of 50 mm (AC254-050-C, Thorlabs) was used as the objective. The beam size on the objective lens was measured to be ∼5.0 mm, yielding an effective numerical aperture (NA) of ∼0.05. The depth of field (DOF) of the OCT system was calculated to be ∼500 *μ*m. Above the objective, a wedge (PS814-C, Thorlabs) was used to adjust the incident angle of the beam on the sample to minimize surface reflection from the latex. OCT signals were generated by interfering reflected light from the reference mirror with that from the latex. Interference signals were detected by a custom spectrometer which consists of a diffraction grating, an F-theta lens (FTH100-1064, Thorlabs) and a line-scan camera (SU1024LDH2, Sensors Unlimited, USA), operating at an axial scan (A-scan) rate of 20.7 kHz. The maximum imaging depth of the OCT system was measured to be 2.2 mm in the latex, considering using the refractive index value of polystyrene (n = 1.57). The axial resolution of the system was measured to be ∼4.3 *μ*m in the latex, and the transverse resolution was measured to be ∼14 *μ*m. The sensitivity of the OCT system was measured to be ∼101 dB.

Besides OCT imaging, OCT-Gravimetry-Video method can perform gravimetric measurement and video recording. The gravimetric measurement was achieved using a digital balance with a 0.001 g precision (AD60, Torbal, USA). 5.7 g latex was weighed and spread uniformly onto a Petri dish (∼8.5 cm in diameter, Kord-Valmark, USA) so that the initial wet latex thickness was ∼1 mm. A video camera (HERO Session, GoPro, USA) was used to capture the top-view time-lapse photos of the latex under white light. A hygrometer/thermometer (TH0165, Perfect Prime, USA) was installed nearby to track changes of environment’s temperature and humidity. All setups were connected and controlled by the computer. All measurements were synchronized so that data measured from all setups were acquired at the same time.

### Data acquisition and processing

#### OCT structural imaging

**Figure 5:**
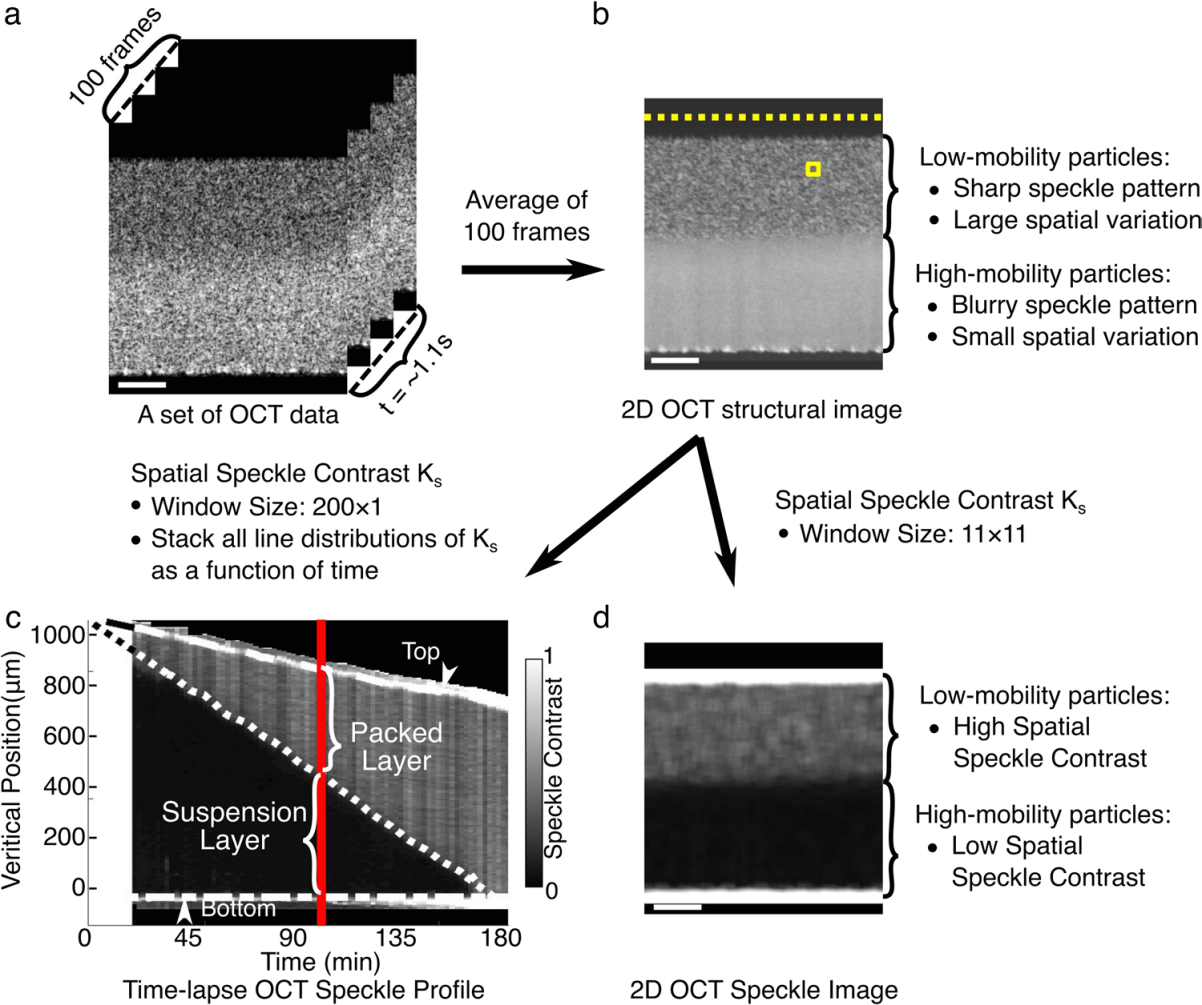
Speckle contrast (*K*_*s*_) analysis of the packing process of L latex. (**a**) 100 consecutive OCT B-scan images of the cross-section of latex in ∼1.1 s, that is ∼11 ms per image. (**b**) 2D OCT structural image, derived by averaging the 100 images in (**a**). (**c**) Time-lapse OCT speckle contrast profile showing the depth-resolved *K*_*s*_ as a function of drying time. *K*_*s*_ is calculated using a 200 × 1 moving window (a yellow dashed line as shown in (**b**)). (**d**) 2D OCT speckle contrast image calculated using an 11 × 11 moving window (a yellow square as shown in (**b**)). Red line in (**c**) indicates the time point (i.e. 98 min) at which the depth-resolved *K*_*s*_ is calculated from (**b**).

OCT imaging was started after the latex was weighed and spread uniformly in the Petri dish. Time-lapse OCT datasets were acquired every 2 min. Each OCT dataset, consisting of 100 repeated B-scans of the latex’s cross-section with a size of 512 × 200 pixels for each B-scan, were acquired in ∼1.1 s. The 200 pixels correspond to the horizontal scanning range of ∼1.0 mm, and the 512 pixels to the vertical imaging depth of ∼2.2 mm. After the time-lapse acquisition, each OCT dataset was processed to generate 100 OCT images (such as Fig. 5a) following a standard protocol for spectral domain OCT^42^. A 2D OCT structural image was generated by averaging all the 100 images of B-scans in each OCT dataset. Then, OCT structural images were flattened according to the Petri dish surface. Image regions above the latex top and below the Petri dish surface were manually removed. Furthermore, by averaging each 2D OCT structural image along the horizontal direction, a time-lapse OCT intensity profile was obtained to show the time-evolution of OCT backscattering intensities in the vertical direction.

#### OCT speckle imaging and determination of domain boundary

We performed speckle contrast analysis^25^ on each 2D OCT structural image averaged from the 100 repeated B-scan images to distinguish the mobility of particles in the latex’s cross-section, in order to characterize its packing process (see Fig. 5). First, we extracted a sub-image for each given pixel of the 2D OCT structural image using the moving window method. For 2D OCT speckle analysis, the moving window contained 11 × 11 pixels. For time-lapse OCT speckle analysis, the window consisted of a row of pixels from the 2D OCT structural image (200 × 1 pixels). Define I_*k*_ as the OCT scattered light intensity of each pixel within the sub-image, where k = 1, 2, …, N, and N was the total number of pixels in each sub-image. Thus, N = 121 for 2D speckle analysis and N = 200 for time-lapse speckle analysis. Then, the average intensity <*I*> and the average intensity square $< {I}^{2} >$ were calculated respectively, as:

3 $$ < I > =\sum _{k=1}^{N}\frac{{I}_{k}}{N}$$

and

4 $$ < {I}^{2} > =\sum _{k=1}^{N}\frac{{I}_{k}^{2}}{N}$$

Then, the standard deviation of intensity for each window was calculated as:

5 $${\sigma }_{s}={\{ < {I}^{2} > -{[ < I > ]}^{2}\}}^{\mathrm{1/2}}$$

Finally, the speckle contrast (*K*_*s*_) in spatial domain for each pixel was calculated as:

6 $${K}_{s}=\frac{{\sigma }_{s}}{ < I > }$$

Looking at the time-lapse B-scan OCT images (see Fig. 5a), the speckle patterns fluctuate with time in the region of particles with high mobility due to Brownian motions; while the speckle patterns show few changes over time in the region of particles with low mobility due to packing of particles. After time averaging (see Fig. 5b), the speckle patterns become blurry in the region with high-mobile particles, while the speckle patterns remain clear and sharp in the region with low-mobile or immobile particles. Therefore, a low speckle contrast (*K*_*s*_, or small spatial variation of light intensity) indicates high mobility of particles, which means that particles remain separately suspended and freely moving in water; while a high *K*_*s*_ (or large spatial variation of light intensity) indicates low mobility of particles, which means that the motions of particles are restricted. By using an 11 × 11 moving window across the time-averaged OCT image (see the yellow square in Fig. 5b), a 2D OCT speckle contrast image is derived to show the distribution of *K*_*s*_ (see Fig. 5d). By using a 200 × 1 moving window (see the yellow dashed line in Fig. 5b), *K*_*s*_ can be calculated to preserve high image resolution in the depth direction (the red line in Fig. 5c); a time-lapse OCT speckle contrast profile can then be derived by stacking the depth-resolved *K*_*s*_ at different times to show the changes of speckle contrast over time (see Fig. 5c).

Based on the differences of speckle contrast, we can distinguish a domain boundary that separates the high *K*_*s*_ region (“packed layer”) and the low *K*_*s*_ region (“suspension layer”), such as those in Fig. 3c (i.e. Fig. 5c) and Figs 3d1–d3. To determine the domain boundary curve in the time-lapse OCT speckle profile, we first calculated the average *K*_*s*_ value of packed layer and that of suspension layer. Then, a threshold *K*_*s*_ value was defined as the mean of both of those *K*_*s*_. The pixels with the same value as the threshold *K*_*s*_ were determined as the packing/suspension domain boundary curve (such as dotted curve in Fig. 3c). After determining the domain boundary in the time-lapse OCT speckle profile, the change of the packed layer thickness over time was obtained (such as the dotted curve in Fig. 3e).

The other way to define domain boundary was based on the differences of scattered light intensity in OCT structural image, since the packed layer and the suspension layer have different scattering properties. Setting the threshold intensity value as the mean of intensities of packed and suspension layers in the time-lapse OCT intensity profile (such as Fig. 3b), the domain boundary curve (such as dotted curve in Fig. 3b) and the curve of packed layer thickness (such as solid curve in Fig. 3e) were determined.

#### Gravimetry and video recording

The total weight of the latex was measured as a function of time (*W*_*latex*_(*t*)), starting from when the latex was transferred into the Petri dish (t = 0). Data was taken every 2 min after the sample was placed on the balance. With a known drying area ($A=\pi {(8.52cm/2)}^{2}=57.01\,c{m}^{2}$), the latex weight per area and the initial latex weight per area are defined as ${w}_{latex}(t)={W}_{latex}(t)/A$ and ${w}_{latex0}={W}_{latex}(t=0)/A$, respectively. Thus, the weight loss per area of the latex, *w*(*t*), was measured as a function of time:

7 $$w(t)={w}_{latex0}-{w}_{latex}(t)$$

then the drying rate was derived as

8 $$Drying\,rate=\frac{dw(t)}{dt}$$

The latex water content, defined as the weight percentage of water in the latex was derived as:

9 $${k}_{w}(t)=(1-\frac{{w}_{latex0}{k}_{s0}}{{w}_{latex0}-w(t)})\times 100 \% $$

For comparison, the drying rate of de-ionized (DI) water (with electrical resistivity of 18.2 MΩ⋅cm at 25 °C) in the Petri dish was measured under the same condition. At the same time, the temperature and humidity of the experimental environment was monitored by a hygrometer/thermometer.

Video recording was initiated before weighing the latex. Time-lapse photos of the Petri dish were recorded every 2 min. The appearance change of the latex was correlated to different drying stages.

## Electronic supplementary material

Supplementary Video 1

Supplementary Video 2

Supplementary Video 3

Supplementary Video 4

Supplementary Video 5

Supplementary materials
