# Supplementary material for: Integrating optical coherence tomography with gravimetric and video analysis (OCT-Gravimetry-Video method) for studying the drying process of polystyrene latex system
Source: Sci Rep. 2018 Aug 28;8:12962. doi: 10.1038/s41598-018-30914-8 (PMC6113323; doi:10.1038/s41598-018-30914-8)
Supplement: Supplementary file 6 — Supplementary materials [file 41598_2018_30914_MOESM6_ESM.pdf]

Supplementary Materials

Integrating optical coherence tomography with gravimetric and video analysis (OCT-Gravimetry-Video method) for studying the drying process of polystyrene latex system

Hao Huang[1,2,+], Yongyang Huang[3,+], Willie Lau[4,5], H. Daniel Ou-Yang[2,6,7,*], Chao Zhou[3,7,*], and Mohamed S. El-Aasser[1,2]

[1]Lehigh University, Department of Chemical and Biomolecular Engineering, Bethlehem, Pennsylvania, 18015, USA

[2]Lehigh University, Emulsion Polymers Institute, Bethlehem, Pennsylvania, 18015, USA

[3]Lehigh University, Department of Electrical and Computer Engineering, Bethlehem, Pennsylvania, 18015, USA

[4]Beijing Oriental Yuhong Waterproof Technology Co., Ltd, Beijing, 100123, China

[5]State Key Laboratory of Special Functional Waterproof Materials, Beijing, 101309, China

[6]Lehigh University, Department of Physics, Bethlehem, Pennsylvania, 18015, USA

[7]Lehigh University, Department of Bioengineering, Bethlehem, Pennsylvania, 18015, USA

[+]These authors contributed equally to this work

[*]Corresponding authors: Chao Zhou (chaozhou@lehigh.edu), and H. Daniel Ou-Yang (hdo0@lehigh.edu)

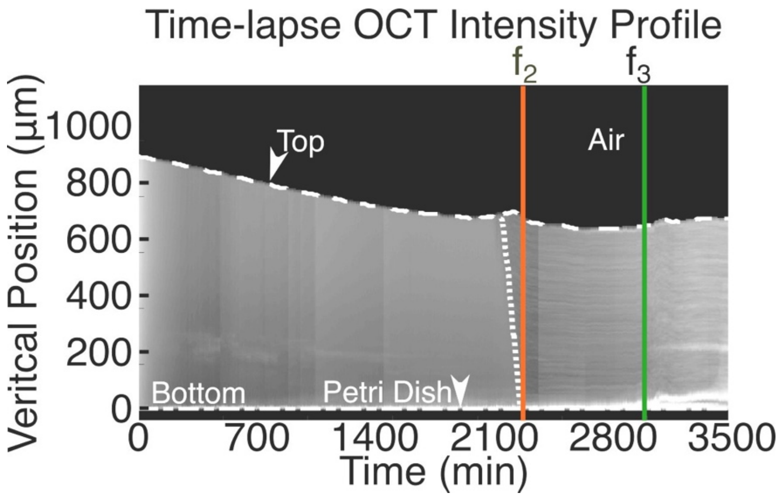

Supplementary Figure 1. Time-lapse OCT intensity profile of the drying process of L latex particles (~125 nm), with the drying rate slowed down and the calculated $\boldsymbol{Pe}$ = 0.4. f2 line denotes the time when the packing process ended (2262 min); f3 line denotes the time when the latex began detachment from Petri dish (2980 min). Note: Fig. 3j in the article is an excerpt between 0 min to 2328 min of this figure.

Supplementary Video 1. OCT-Gravimetry-Video characterization of the drying process of L latex particles (~125 nm). Top left: 2D OCT structural images. Top right: video photos. Bottom left: time-lapse OCT intensity profile. Bottom right: the curves of the global drying rate ($dw(t)/dt$) and the water content in latex ($k_w(t)$).

Supplementary Video 2. OCT-Gravimetry-Video characterization of the drying process of S latex particles (~53 nm). Top left: 2D OCT structural images. Top right: video photos. Bottom left: time-lapse OCT intensity profile. Bottom right: the curves of the global drying rate ($dw(t)/dt$) and the water content in latex ($k_w(t)$).

Supplementary Video 3. Visualization of particles' packing process of L latex using OCT. Top left: 2D OCT structural images. Top right: 2D OCT speckle images. Bottom left: time-lapse OCT intensity profile. Bottom right: time-lapse OCT speckle profile.

Supplementary Video 4. Visualization of particles' packing process of S latex using OCT. Top left: 2D OCT structural images. Top right: 2D OCT speckle images. Bottom left: time-lapse OCT intensity profile. Bottom right: time-lapse OCT speckle profile.

Supplementary Video 5. Visualization of particles' packing process of L latex during the initial 30 min using OCT. Top left: 2D OCT structural images. Top right: 2D OCT speckle images. Bottom left: time-lapse OCT intensity profile. Bottom right: time-lapse OCT speckle profile.
